# The Small Hospitals and the Funds

**Published:** 1904-11-19

**Authors:** 


### HOSPITAL ADMINISTRATION.

### CONSTRUCTION AND ECONOMICS.

### THE SMALL HOSPITALS AND THE FUNDS.

Our contemporary, the *Medical Press*, in an article headed "The Small Hospitals and the Funds" makes certain statements which will not bear investigation. Our contemporary has evidently been misled by general assertions which have little or no substance in fact. The contention of the writer of the article in the *Medical Press* of the 9th instant is in effect that "the chief cause of the decline of the small hospitals has in all probability been the policy of the Sunday and the King Edward's Hospital Funds, which, whether intentionally or otherwise, has resulted in the withholding of grants and injury to the reputation of a host of small but deserving medical charities." We have been unable to find this "host of small but deserving medical charities" referred to by the *Medical Press*, and venture to ask our contemporary to supply the names and addresses of the institutions in question, which we have not been able to discover though we have carefully searched for evidence of their existence in fact. We have never concealed our own belief that in the present day small private adventure hospitals are very undesirable things. We have frequently declared that it should be made impossible by law to establish any such institution in a large town without the license of the County Council, or some equally representative body of independent people, who are in a position to test the bona fides of the promoters, and to judge whether the proposed small hospital is wanted or even desirable. We cannot suppose that the *Medical Press* can be an advocate for such private adventure hospitals and, if it is not, our contemporary, as we have said, has evidently been misled as to the facts.

In the matter of grants, too, both the Hospital Sunday and the King's Hospital Funds appear to err, if they err at all, in giving to too great a number of small hospitals. For instance, the Hospital Sunday Fund, in addition to the grants made in 1904 to 31 general hospitals and 28 special hospitals, including 5 chest hospitals, 17 children's hospitals, and 6 lying-in hospitals, made grants to 33 other special hospitals, including 6 hospitals for women, and also to 22 cottage hospitals.

The contention of the *Medical Press* is that in cases where grants have been made to special hospitals they have been altogether inadequate, and have shown a tendency to decrease, as we understand the argument. What are the facts? During the five years, 1900-1904 inclusive, the Hospital Sunday Fund, has made an average grant of £7,112 to these 33 small special hospitals. During the year 1904 the annual amount distributed by the Sunday Fund amongst these institutions was £7,755, or upwards of £600 above the average. The figures for the King's Fund for 1904 are not available, but in the case of the same 33 hospitals the King's Fund made grants in 1900 to the extent of £5,600, whilst in 1903 the grants made to these institutions amounted to £15,000. In other words, the contributions from the King's Fund to the smaller hospitals has increased during the last four years to the extent of nearly three times the sum they formerly received. Indeed, the grants made to them must, in fact, represent additional contributions, and consequently increased resources for the distribution of medical aid to the sick poor of the metropolis. In view of the facts here given it must be evident that the statements made by the writer in the *Medical Press* are very wide of the mark, a fact which our contemporary must be the first to admit. We would also urge our contemporary, in the interests of the public, to immediately publish a complete list of the "host of small but deserving medical charities" stated to have been injured by the establishment of, and the methods of distribution pursued by, the Sunday and King's Hospital Funds.

